# Supplementary material for: Distractibility and impulsivity neural states are distinct from selective attention and modulate the implementation of spatial attention
Source: Nat Commun. 2022 Aug 15;13:4796. doi: 10.1038/s41467-022-32385-y (PMC9378734; doi:10.1038/s41467-022-32385-y)
Supplement: Supplementary file 1 — Supplementary Information [file 41467_2022_32385_MOESM1_ESM.pdf]

Supplementary information file

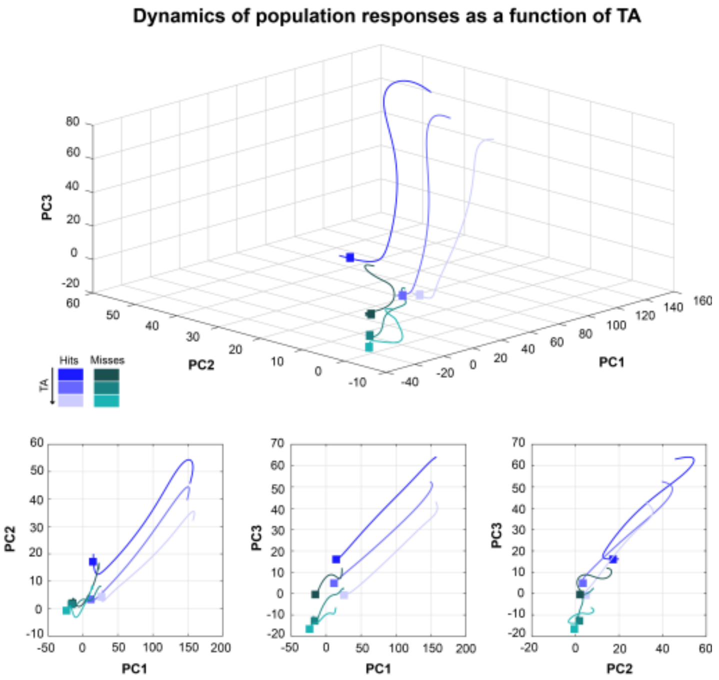

Supplementary Figure 1. PCA analysis as a function of the position of attention and the upcoming behavioral outcome. Projection onto the three first principal components of the MUA activity pooled by upcoming behavioral outcome (hit trials blue shades, miss trials green shades) and TA (darker tones corresponding to smaller TA values) during the 300 to 0 ms preceding target presentation, locked to target onset. MUA neuronal activity cumulated over all sessions.

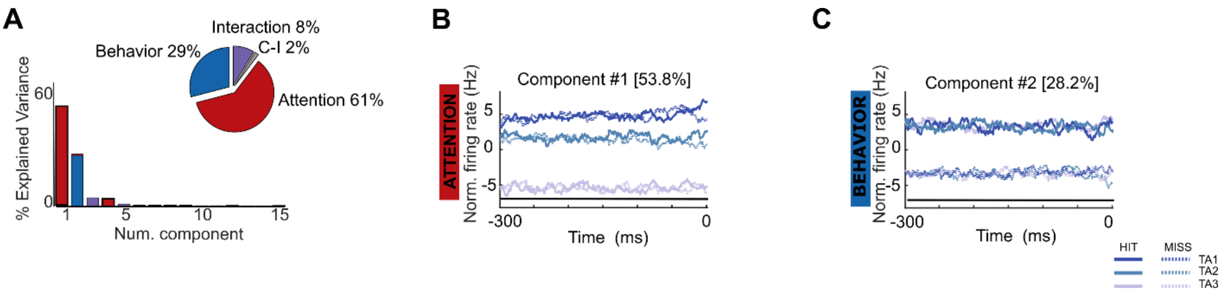

Supplementary Figure 2. At single session level, dPCA unmixes variance between attention to target distance and behavior outcome. (A) Distribution of the variance explained between the two parameters (attention to target distance and upcoming target behavior) for a specific session. (B and C) MUA activity from the six different conditions (hit or miss trials for each of the three TA bins) projected on the component that maximally explains attention to target distance (B) and upcoming target behavior (C).

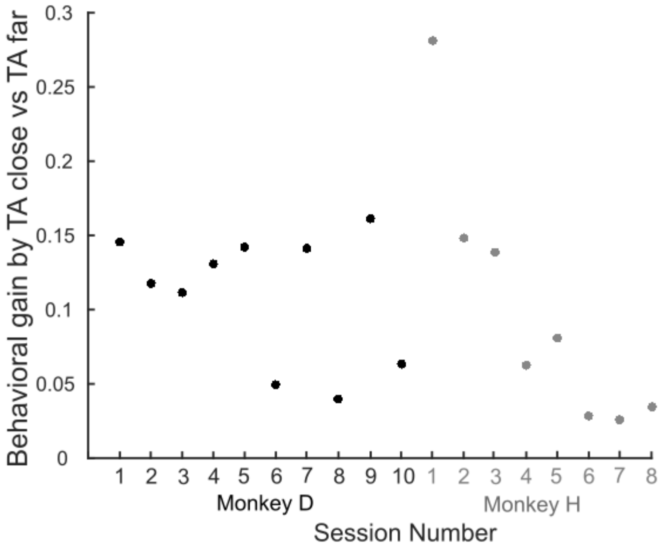

Supplementary Figure 3. Behavioral gain (hit rate) produced by an allocation of attention close (trials with TA close) to relative to far (trials with TA far) from upcoming target location, for each monkey and each session (N = 18 sessions).

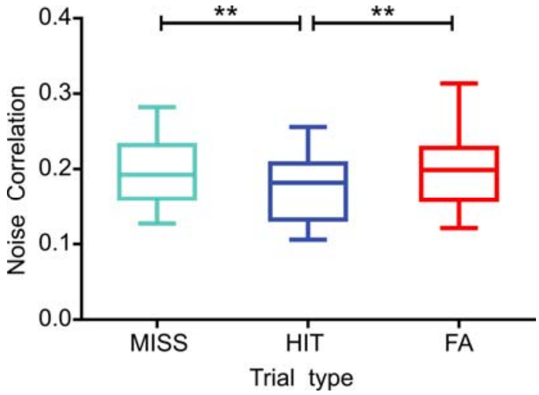

Supplementary Figure 4. Boxplot representing the distribution of noise correlation across sessions for hit trials (blue), miss trial (green) and false alarm trials (red), after equalizing the mean TA value between trial types (Two-sided Wilcoxon signed rank test, ** $p<0.01$). Lower and upper box boundaries in boxplots reflect the 90th and 10th percentiles, respectively, line inside box reflects the median and lower and upper error lines show the min and max value of each distribution, respectively.

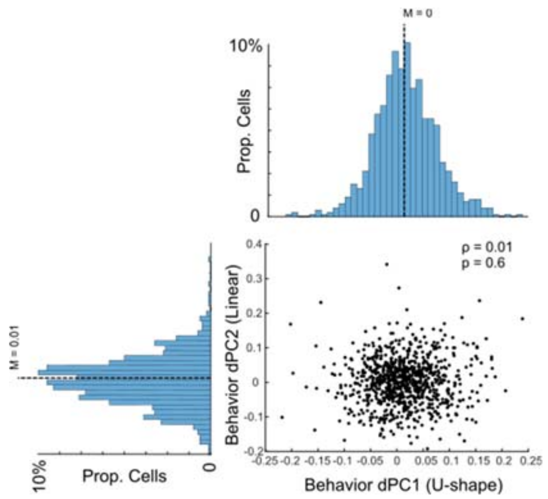

Supplementary Figure 5. For each neuron, we use the first (U-shape) and second (Linear) demixed PCs described in figure 8 to plot its location on the plane defined by these two components. These components present a weight distribution that tends to be centered and equally distributed around zero (cf. respective histograms). The scatterplot shows the relationship between the neurons' weights in the dPC1 and dPC2 demixed components (N = 848 neurons). This correlation is non-significant (Spearman Correlation, $\rho = 0.01$, $p=0.6$). The dot product between these components indicates that these components are orthogonal (89 degrees).

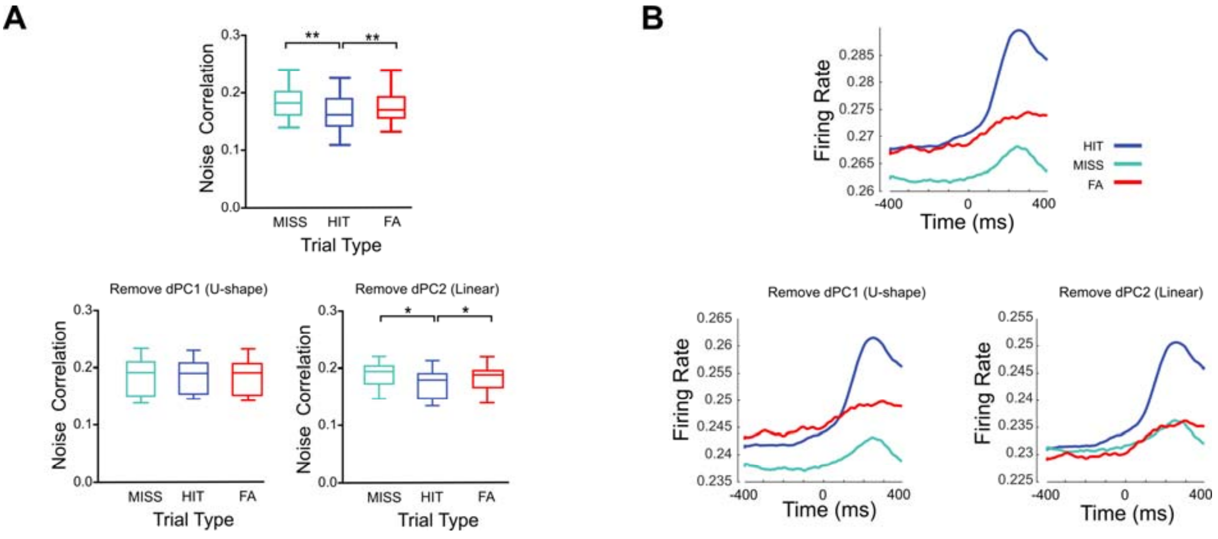

Supplementary Figure 6 A. *(Up)* Boxplot of noise correlations across sessions, during a pre-target interval (-300 to 0 ms), for each of the three trial types: Hit (blue), Miss (green) and false alarm trials (red). *(Bottom)* Boxplot of noise correlations across sessions from data reconstructed after removing the variance explained by dPC1 (U-shape, Left), or dPC2 (Linear, Right). (Two sided Wilcoxon signed rank test, * p<0.05, ** p<0.01). **B.** *(Up)* Mean firing rates recorded during Hit (blue), Miss (green) and false alarm trials (red) locked to target onset (interval -400 to 400 ms). *(Bottom)* Mean firing rates averaged across similar trial types, after removing the variance explained by dPC1 (U-shape, Left), or dPC2 (Linear, Right). Lower and upper box boundaries in boxplots reflect the 90th and 10th percentiles, respectively, line inside box reflects the median and lower and upper error lines show the min and max value of each distribution, respectively.

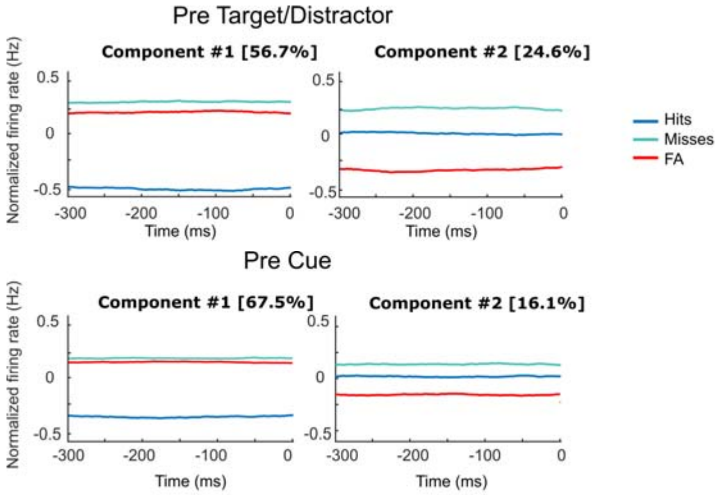

Supplementary Figure 7. dPCA unmixes variance associated to trial type (Hit, Miss and FA) in two independent components. All as in figure 8, except for MUA activities being cumulated over all sessions.

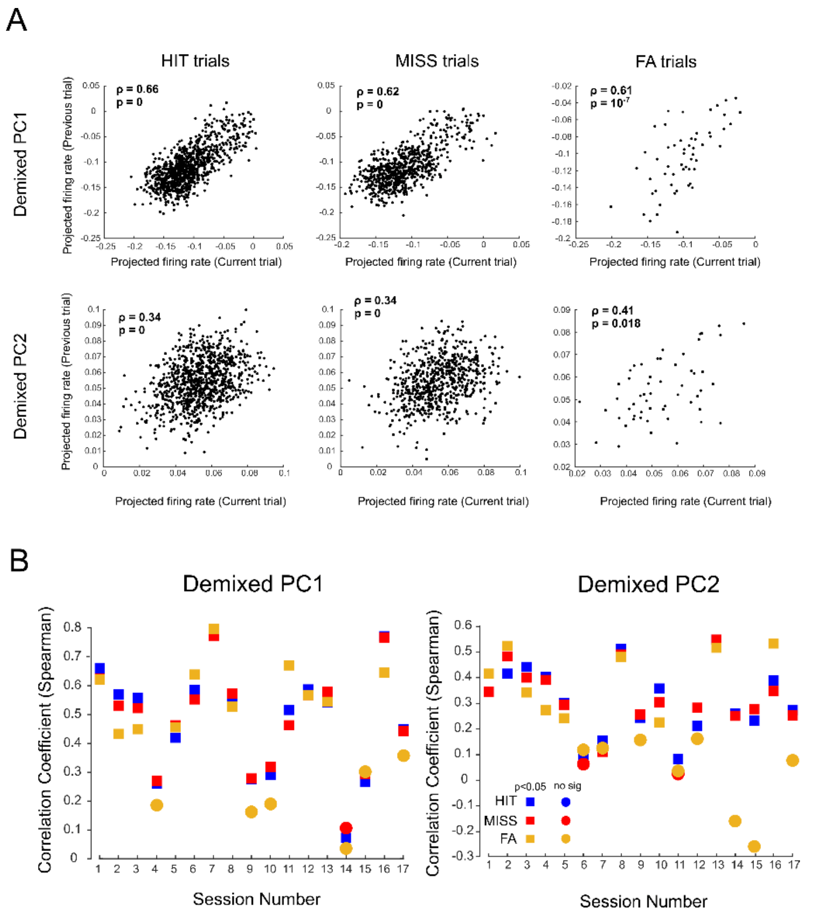

Supplementary Figure 8. (A) Scatter plot representing the distribution of the pre-cue firing rate projected onto dPC1 (U-shape) and dPC2 (Linear) of the current trial (for hit trials, miss trials and false alarm trials) with the projected firing rates obtained from the previous trial (irrespective of the trial type) for one representative session. **(B)** Spearman correlation coefficients of the same correlations performed in (A) for all sessions, per trial type (current trial, hit (blue), miss (red) and false alarm (yellow) responses; squares. Spearman correlation $p<0.05$; circles, $p>0.05$).
