# Supplementary material for: Distractibility and impulsivity neural states are distinct from selective attention and modulate the implementation of spatial attention
Source: Nat Commun. 2022 Aug 15;13:4796. doi: 10.1038/s41467-022-32385-y (PMC9378734; doi:10.1038/s41467-022-32385-y)
Supplement: Supplementary file 3 — Reporting Summary [file 41467_2022_32385_MOESM3_ESM.pdf]

nature portfolio

Corresponding author(s): Suliann Ben Hamed, PhD

Last updated by author(s): 2022/06/27

Reporting Summary

Nature Portfolio wishes to improve the reproducibility of the work that we publish. This form provides structure for consistency and transparency in reporting. For further information on Nature Portfolio policies, see our Editorial Policies and the Editorial Policy Checklist.

Statistics

For all statistical analyses, confirm that the following items are present in the figure legend, table legend, main text, or Methods section.

n/a	Confirmed	
☐	☒	The exact sample size (*n*) for each experimental group/condition, given as a discrete number and unit of measurement
☐	☒	A statement on whether measurements were taken from distinct samples or whether the same sample was measured repeatedly
☐	☒	The statistical test(s) used AND whether they are one- or two-sided<br>*Only common tests should be described solely by name; describe more complex techniques in the Methods section.*
☐	☒	A description of all covariates tested
☐	☒	A description of any assumptions or corrections, such as tests of normality and adjustment for multiple comparisons
☐	☒	A full description of the statistical parameters including central tendency (e.g. means) or other basic estimates (e.g. regression coefficient) AND variation (e.g. standard deviation) or associated estimates of uncertainty (e.g. confidence intervals)
☐	☒	For null hypothesis testing, the test statistic (e.g. F, t, r) with confidence intervals, effect sizes, degrees of freedom and P value noted<br>*Give P values as exact values whenever suitable.*
☒	☐	For Bayesian analysis, information on the choice of priors and Markov chain Monte Carlo settings
☒	☐	For hierarchical and complex designs, identification of the appropriate level for tests and full reporting of outcomes
☐	☒	Estimates of effect sizes (e.g. Cohen's d, Pearson's r), indicating how they were calculated

Our web collection on statistics for biologists contains articles on many of the points above.

Software and code

Policy information about availability of computer code

Data collection	Data was collected using Plexon Omniplex® neuronal data acquisition system
Data analysis	Data were analyzed using MATLAB R2015b © (The Mathworks Inc., Natick, Massachusetts)

For manuscripts utilizing custom algorithms or software that are central to the research but not yet described in published literature, software must be made available to editors and reviewers. We strongly encourage code deposition in a community repository (e.g. GitHub). See the Nature Portfolio guidelines for submitting code & software for further information.

Data

Policy information about availability of data

All manuscripts must include a data availability statement. This statement should provide the following information, where applicable:

- Accession codes, unique identifiers, or web links for publicly available datasets
- A description of any restrictions on data availability
- For clinical datasets or third party data, please ensure that the statement adheres to our policy

The data that support the findings of this study are available from the corresponding author upon reasonable request. Data are still being analyzed for other purposes and cannot be made publically available at this time. Source data are provided with this paper.

Human research participants

Policy information about studies involving human research participants and Sex and Gender in Research.

Reporting on sex and gender	Not Applicable
Population characteristics	Not Applicable
Recruitment	Not Applicable
Ethics oversight	Not Applicable

Note that full information on the approval of the study protocol must also be provided in the manuscript.

Field-specific reporting

Please select the one below that is the best fit for your research. If you are not sure, read the appropriate sections before making your selection.

☒ Life sciences ☐ Behavioural & social sciences ☐ Ecological, evolutionary & environmental sciences

For a reference copy of the document with all sections, see nature.com/documents/nr-reporting-summary-flat.pdf

Life sciences study design

All studies must disclose on these points even when the disclosure is negative.

Sample size	In non-human primate experiments, sample sizes in single group studies are typically set to two since they are limited by both ethical and practical constraints (Bliss-Moreau et al, 2021). In addition, a huge number of studies (most of them very relevant in the field) include two monkeys (Astrand et al, 2014; Rigotti et al, 2013, Mante et al, 2013; Fiebelkorn et al, 2019)
Data exclusions	No data were excluded in the analysis
Replication	Results were performed at whole population level and at recording session level. In both approaches, the results were qualitatively similar.
Randomization	Randomization does not apply in this study, as only one group of two monkeys was included.
Blinding	The monkeys name were codified, and the researcher conducting the analysis had no knowledge of the monkey's identity when analyzing the neuronal data.

Reporting for specific materials, systems and methods

We require information from authors about some types of materials, experimental systems and methods used in many studies. Here, indicate whether each material, system or method listed is relevant to your study. If you are not sure if a list item applies to your research, read the appropriate section before selecting a response.

Materials & experimental systems

n/a	Involved in the study	
☒	☐	Antibodies
☒	☐	Eukaryotic cell lines
☒	☐	Palaeontology and archaeology
☐	☒	Animals and other organisms
☒	☐	Clinical data
☒	☐	Dual use research of concern

Methods

n/a	Involved in the study	
☒	☐	ChIP-seq
☒	☐	Flow cytometry
☒	☐	MRI-based neuroimaging

Animals and other research organisms

Policy information about studies involving animals; ARRIVE guidelines recommended for reporting animal research, and Sex and Gender in Research

Laboratory animals	Macaque mulatta (6 years and 7 years old)

Wild animals	This study did not involve wild animals
Reporting on sex	Two male macaques were used in this study
Field-collected samples	This study did not involve samples collected in the field
Ethics oversight	All surgical and experimental procedures were approved by the local animal care committee (C2EA42-13-02-0401-01) approved by the french Ministry of Research and in compliance with the European Community Council, Directive 2010/63/UE on Animal Care.

Note that full information on the approval of the study protocol must also be provided in the manuscript.
